# A Randomized Controlled Trial on the Therapeutic Effect of Aloe Vera Extract on Diabetic Foot Ulcers

**DOI:** 10.7759/cureus.88956

**Published:** 2025-07-29

**Authors:** Sandhiya T, Sahasyaa Adalarasan, Samyuktha U, Suganeshwari MD, Ramya R

**Affiliations:** 1 Medicine School, Madras Medical College, Chennai, IND; 2 Institute of Pharmacology, Madras Medical College, Chennai, IND; 3 Department of Pathology, Sudha Saseendharan Siddha Medical College, Kaliyakkavilai, IND

**Keywords:** aloe vera extract, bates jensen wound assessment tool (bwat) score, case control studies, diabetic foot ulcers management, diabetic mellitus

## Abstract

Introduction

Diabetes mellitus is a chronic metabolic disorder characterized by hyperglycemia resulting from defects in insulin secretion, insulin action, or both. Among its complications, diabetic foot ulcers (DFUs) pose a significant healthcare challenge which result from impaired healing and infection risks, demanding better therapies. The aloe vera extract promotes wound healing by enhancing tissue regeneration and offering antimicrobial effects. This study evaluates its therapeutic effect on DFUs through a randomized controlled trial.

Methods

The study involved 60 patients sampled and followed up between May 1, 2025, and June 15, 2025. It compared wound healing outcomes between two groups selected by the lottery method. A control group (30 patients) receiving standard care and a blinded study group (30 patients) treated with aloe vera extract were assessed using the Bates-Jensen Wound Assessment Tool (BWAT). Wound healing was monitored on days 0, 14, and 28, and data were analyzed for significant differences.

Results

The study's demographic data showed comparable baseline characteristics between groups, with no adverse effects from aloe vera use. BWAT scores were recorded on days 0, 14, and 28, and the average scores for the case group and the control group, respectively, were 42±3.11 and 43±3.58 on day 0, 31±2.16 and 35±2.61 on day 14, 19±2.17 and 25±2.17 on day 28. Average scores decreased more rapidly in the case group than in the control group. A chi-square test confirmed a significant association between aloe vera treatment and enhanced wound healing (p = 0.0029).

Conclusion

This single-blinded randomized controlled trial shows that topical aloe vera significantly improves DFU healing compared to standard care. Its effects are likely due to the promotion of angiogenesis, reduction in inflammation, and antimicrobial action. Aloe vera is a safe, affordable option, especially for low-resource settings. Larger studies with longer follow-up are needed to confirm and expand its clinical use.

## Introduction

Background and rationale

Diabetes mellitus is a chronic metabolic disorder characterized by hyperglycemia resulting from defects in insulin secretion, insulin action, or both. Among its complications, diabetic foot ulcers (DFUs) pose a significant healthcare challenge, particularly in the context of the rising global prevalence of diabetes [1]. DFUs are associated with prolonged wound healing, increased risk of infection, reduced quality of life, and high healthcare costs. The complexity of wound healing in diabetic patients necessitates the exploration of adjunctive therapeutic strategies.

Aloe vera extract has gained attention for its potential wound-healing properties. It has been shown to promote glycosaminoglycan synthesis and epithelial regeneration [2], while also enhancing the expression of integrins, platelet endothelial cell adhesion molecule-1 (PECAM-1), and transforming growth factor beta (TGF-β), which are critical mediators of tissue repair in preclinical studies [3]. Its antimicrobial properties further support its potential role in DFU management by reducing microbial load and preventing secondary infections [4].

Despite encouraging preclinical data, clinical evidence supporting the efficacy of aloe vera in DFU management remains sparse, with few well-designed randomized controlled trials evaluating its effects in human subjects. This study was conducted to address this gap.

Objective

The objective of this randomized controlled trial (RCT) is to evaluate the therapeutic effect of topical aloe vera extract on DFUs, with a focus on wound healing outcomes and safety. The study aims to determine whether aloe vera can serve as a safe and effective adjunct to standard care and whether its use results in clinically meaningful improvements in wound healing without adverse effects.

## Materials and methods

Ethical approval and trial registration

The proposal for the present study was submitted to the Institutional Ethics Committee at Madras Medical College, Chennai, India, and received approval before the commencement of the research (approval number: 61022024, dated February 4, 2025). The study was registered under the Clinical Trials Registry of India (CTRI/2025/04/085953, dated April 29, 2025).

Trial design and setting

This was a single-center, prospective, parallel-group RCT with an allocation ratio of 1:1, designed under a superiority framework. The study was conducted in the surgical wards of Rajiv Gandhi Government General Hospital, a tertiary care government hospital in Chennai, India. The trial duration spanned from May 1, 2025, to June 15, 2025.

Participant eligibility

Patients included in the study were those diagnosed with type 2 diabetes mellitus who presented with DFUs requiring debridement. Patients were excluded if they had peripheral arterial disease, as confirmed by Doppler ultrasound using a linear array probe, or if they had evidence of osteomyelitis. Additional exclusion criteria included poor glycemic control despite insulin therapy, pregnancy or lactation, and the presence of hepatic or renal failure. 

Interventions

Participants in the control group received standard care, including surgical debridement when indicated and daily dressing with povidone-iodine gauze. Debridement was performed every two to three days when necrotic tissue was observed. Antibiotic therapy was selected based on the hospital antibiogram, either intravenous piperacillin-tazobactam (4.5 g every eight hours) or intravenous ceftriaxone (2 g twice daily).

Participants in the study group received the same standard care, with the addition of topical application of a 100% w/w aloe vera gel extract (commercially obtained, glycerin-based for stability). The gel was applied post-debridement in a dose dependent on wound extent, prior to dressing.

Outcomes

The primary outcome was wound healing as assessed by the Bates-Jensen Wound Assessment Tool (BWAT) [5]. The BWAT evaluates parameters such as size, depth, tissue type, and exudate, with scores ranging from 13 (best) to 65 (worst). Higher scores indicate greater severity. The BWAT score was measured on days 0, 14, and 28. Special focus was given to depth, epithelialization, and necrotic tissue. Permission for tool use was obtained from the creator of the tool. No secondary outcomes or harms were pre-specified. No adverse events were reported.

Sample size

The sample size was calculated using the formula for comparing two means, assuming an alpha error of 0.05, a power of 80%, and a confidence interval (CI) of 95%. The required sample size was 27 participants per group. Accounting for a 10% dropout rate, this was rounded up to 30 participants per group (N=60).

Randomization

Randomization was done using a simple lottery method. Sequence generation was generated manually via sealed slips. Allocation was concealed using sequentially numbered, opaque, sealed envelopes (SNOSE), which were opened only after participant enrollment. The sequence was generated and implemented by two independent researchers not involved in patient care or outcome assessment to maintain allocation concealment.

Blinding

Due to the nature of the intervention, blinding of participants and care providers was not feasible. However, outcome assessors were blinded to group allocation to reduce assessment bias.

Statistical methods

Data analysis was conducted using IBM SPSS Statistics for Windows, version 24 (IBM Corp., Armonk, New York, United States). Descriptive statistics (mean, SD) were used to summarize data. For between-group comparisons, chi-square tests were used to evaluate categorical outcomes (such as the proportion of patients achieving a ≥22-point reduction in BWAT score, defined post hoc). No imputation for missing data was necessary as all participants completed follow-up. No additional subgroup or sensitivity analyses were conducted.

Changes to trial protocol

No changes were made to the study protocol after trial commencement.

## Results

Participant flow and recruitment

**Figure 1:**
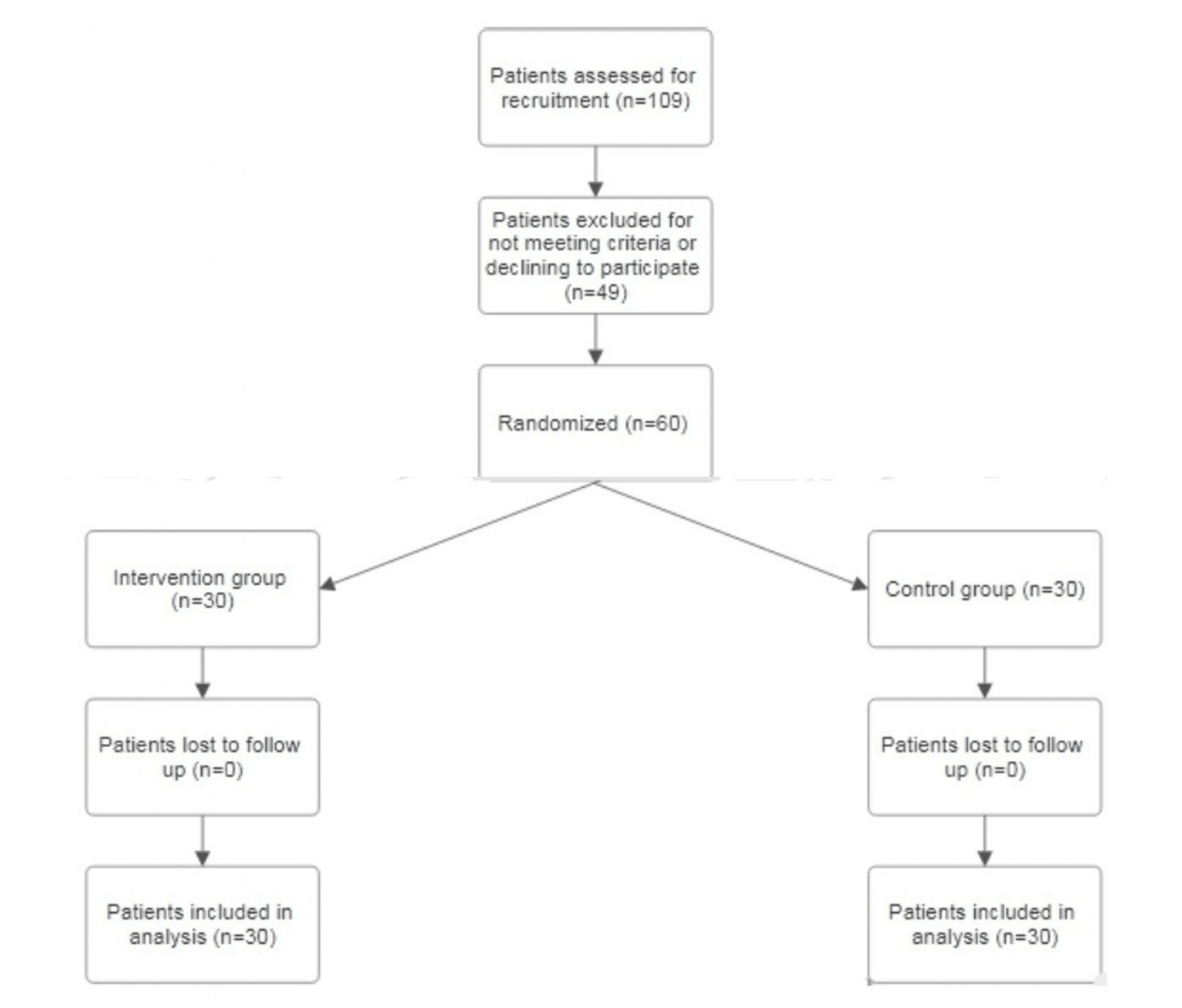
CONSORT 2025 flowchart CONSORT: Consolidated Standards of Reporting Trials

A total of 109 participants were assessed between May 1, 2025, and June 15, 2025, out of which 60 met the eligibility criteria. All participants who met the eligibility criteria were randomly assigned to one of two groups: the aloe vera treatment group (n = 30) and the control group (n = 30). No participants were lost to follow-up or excluded after randomization. All 60 participants completed the study and were included in the final analysis (Figure 1). There were no early terminations or trial stoppages.

Intervention and comparator delivery

All participants in the control group received standard care, which included debridement as needed and daily povidone-iodine gauze dressing. In the treatment group, aloe vera extract was applied topically post-debridement before dressing. Both groups also received antibiotics guided by the hospital antibiogram: either IV piperacillin-tazobactam (4.5 g every eight hours) or IV ceftriaxone (2 g twice daily). The interventions were delivered by trained surgical residents, and adherence to the protocol was monitored throughout. No deviations were reported.

Baseline data

Table 1 summarizes the baseline demographic and clinical characteristics of both groups. The groups were comparable in terms of age, gender distribution, capillary blood glucose (CBG) levels, and initial BWAT scores.

**Table 1:** Demographic characteristics of the study population CBG: capillary blood glucose

	Age (years), mean±SD	Male gender, n (%)	CBG at time of admission (in mg/dL), mean±SD	Adverse effects reported, n (%)	Average BWAT score at enrolment
Case	50.87 ± 9.26	14 (46.67%)	263.41 ± 68.62	None (0%)	42.3
Control	55.07 ± 13.56	16 (53.34%)	259.11 ± 53.73	NA	43.1

Outcomes and estimation

The primary outcome was wound healing, assessed using the Bates-Jensen Wound Assessment Tool (BWAT). Mean BWAT scores were recorded on days 0, 14, and 28 for both groups (Table 2).

**Table 2:** Average BWAT scores of the two groups on days 0, 14, 28 BWAT: Bates-Jensen Wound Assessment Tool

	Day 0, mean±SD	Day 14, mean±SD	Day 28, mean±SD
Case	42 ± 3.11	31 ± 2.16	19 ± 2.17
Control	43 ± 3.58	35 ± 2.61	25 ± 2.17

To further evaluate outcomes, participants were categorized based on their improvement: those with a ≥22-point reduction in BWAT score were classified as having "high improvement." This cutoff was not pre-specified but determined post hoc based on the observed distribution of score changes. It closely approximated the median reduction in the case group and clearly distinguished it from the control group. A chi-square test showed a significant association between aloe vera treatment and high improvement (p = 0.0029) (Table 3). 

**Table 3:** Chi-Square table

Group	High improvement (≥ 22 point drop)	Low improvement (≥ 22 point drop)
Case	13	2
Control	1	14
Total	14	16

Harms

No adverse effects were reported in the aloe vera group, either spontaneously by participants or upon active questioning. There were no unintended events in either group.

Ancillary analyses

No subgroup or sensitivity analyses were performed. All analyses presented were post hoc unless otherwise specified.

## Discussion

Interpretation

Aloe vera promotes wound healing primarily by enhancing the production of extracellular matrix components, particularly glycosaminoglycans, which play a crucial role in maintaining tissue hydration and facilitating cellular signaling in rats [6]. This mechanism is consistent with previous in vivo studies on dermal regeneration. Further studies in humans are warranted to confirm this, but it is also reasonable to expect a similar effect in human tissue.

Additionally, aloe vera stimulates the expression of key growth factors such as TGF-β and endothelial markers like PECAM1, thereby promoting neovascularization and re-epithelialization [7]. These effects align with the accelerated healing observed in our study.

Another important attribute of aloe vera is its antimicrobial activity. By inhibiting the growth of both gram-positive and gram-negative bacteria, it helps reduce the microbial burden in chronic wounds, thereby supporting uninterrupted tissue repair [8]. This is especially relevant in the context of DFUs, where persistent infections can significantly delay healing.

Furthermore, aloe vera has demonstrated anti-inflammatory and antioxidant effects. It modulates inflammatory pathways and reduces oxidative stress at the wound site, facilitating tissue regeneration and providing symptomatic relief such as reduced pain and swelling [9]. A recent systematic review including more than 100 RCTs and other studies has comprehensively summarized these therapeutic properties of aloe vera [10]. These mechanisms support the findings of the present study, wherein patients exhibited accelerated and more complete wound healing trajectories.

Experimental data further corroborate these effects. An animal study reported a statistically significant increase in glycosaminoglycan content (p < 0.0001) in the aloe vera-treated group, affirming its role in wound repair [11]. Similarly, a clinical study conducted in Pakistan found aloe vera dressings to be more effective than normal saline in promoting healing [12]. Another investigation employing the BWAT demonstrated a more substantial reduction in wound scores with aloe vera (35.1 vs. 31.42) [13]. Studies involving more frequent applications, such as twice-daily interventions, also yielded comparable results [14]. Novel therapies, such as placenta-derived mesenchymal stem cell hydrogels, are currently being explored for DFU management, highlighting the growing interest in biologically-based treatments [15].

Limitations

The primary limitation of the present study is its small sample size, as it serves as a pilot investigation into the post-debridement application of aloe vera in a population that has not been widely studied. Patient compliance to their oral hypoglycemic medication and factors like wound care could be potential confounding variables in the present study.

Additionally, the study was limited to a single center and used only one type of wound dressing intervention. The generalizability of the results to broader populations or different clinical settings may thus be restricted. No double-blinding was done, and subjective components of the BWAT scoring may be influenced by observer bias.

## Conclusions

This RCT demonstrates that topical application of aloe vera extract significantly enhances the healing of DFUs compared to standard care alone. Given its pro-angiogenic, anti-inflammatory, and antimicrobial properties, with its safety, affordability, and ease of use, aloe vera gel holds promise as an adjunctive therapy. This is particularly in post-debridement, in DFU management protocols at low-resource settings, where advanced wound care options may be limited.

To incorporate this into routine clinical practice, its efficacy must be validated through large-scale, multicenter randomized trials with standardized application protocols, including dosage, frequency, and duration of use. Future research should also explore comparative effectiveness with other advanced wound therapies and cost-effectiveness analyses to support broader implementation. These steps are essential to establish a robust evidence base for its inclusion in clinical guidelines.
